# Mode of gene action and heterosis for physiological, biochemical, and agronomic traits in some diverse rice genotypes under normal and drought conditions

**DOI:** 10.3389/fpls.2023.1108977

**Published:** 2023-03-31

**Authors:** Mohamed S. Abd-El-Aty, Mahmoud I. Abo-Youssef, Mohamed M. Bahgt, Omar M. Ibrahim, Hana Faltakh, Hela Nouri, Shereen Magdy Korany, Emad A. Alsherif, Hamada AbdElgawad, Amira M. El-Tahan

**Affiliations:** ^1^ Agronomy Department, Faculty of Agriculture, Kafr El-Sheikh University, Kafr El-Sheikh, Egypt; ^2^ Rice Research and Training Center, Field Crops Research Institute, Agricultural Research Center, Egypt; ^3^ Plant Production Department, Arid Lands Cultivation Research Institute, The City of Scientific Research and Technological Applications, SRTA-City, Borg El Arab, Alexandria, Egypt; ^4^ Department of Basic Sciences, Deanship of Preparatory Year and Supporting Studies, Imam Abdulrahman Bin Faisal University, P.O. Box 1982, Dammam, 34212, Saudi Arabia; ^5^ Department of Biology, College of Science, Princess Nourah bint Abdulrahman University, P.O. Box 84428, Riyadh 11671, Saudi Arabia; ^6^ Biology Department, College of Science and Arts at Khulis, University of Jeddah, Jeddah 21959, Saudi Arabia; ^7^ Botany and Microbiology Department, Faculty of Science, Beni-Suef University, Beni‒Suef 62521, Egypt

**Keywords:** rice, line x tester, gene action, heterosis, combing ability, water deficit, physiological and biochemical traits

## Abstract

Water scarcity is a crucial environmental stress that constrains rice growth and production. Thus, breeding for developing high-yielding and drought-tolerant rice genotypes is decisive in sustaining rice production and ensuring global food security, particularly under stress conditions. To this end, this study was conducted to evaluate the effects of water deficit on 31 genotypes of rice (seven lines, viz., Puebla, Hispagran, IET1444, WAB1573, Giza177, Sakha101, and Sakha105, and three testers, viz., Sakha106, Sakha107, and Sakha108) and their 21 crosses produced by line × tester mating design under normal and water deficit conditions; this was to estimate the combining ability, heterosis, and gene action for some traits of physiological, biochemical, and yield components. This study was performed during the summer seasons of 2017 and 2018. The results showed that water deficit significantly decreased relative water content, total chlorophyll content, grain yield, and several yield attributes. However, osmolyte (proline) content and antioxidant enzyme activities (CAT and APX) were significantly increased compared with the control condition. Significant mean squares were recorded for the genotypes and their partitions under control and stress conditions, except for total chlorophyll under normal irrigation. Significant differences were also detected among the lines, testers, and line × tester for all the studied traits under both irrigation conditions. The value of the σ²GCA variance was less than the value of the σ²SCA variance for all the studied traits. In addition, the dominance genetic variance (σ^2^D) was greater than the additive genetic variance (σ^2^A) in controlling the inheritance of all the studied traits under both irrigation conditions; this reveals that the non-additive gene effects played a significant role in the genetic expression of the studied traits. The two parental genotypes (Puebla and Hispagran) were identified as good combiners for most physiological and biochemical traits, earliness, shortness, grain yield, and 1,000-grains weight traits. Additionally, the cross combinations Puebla × Sakha107, Hispagran × Sakha108, and Giza177 × Sakha107 were the most promising. These results demonstrated the substantial and desirable specific combining ability effects on all the studied traits, which suggested that it could be considered for use in rice hybrid breeding programs.

## 1 Introduction

Rice (*Oryza sativa* L.) is a self-pollinated cereal crop belonging to the family *Poaceae* with the chromosome number 2*n* = 24 (Al Azzawi et al., 2020; Kumar et al., 2020). It is a major food crop and the most widely produced cereal crop in tropical and subtropical regions worldwide. Rice is also the most widely consumed staple food for many of the world’s population (Samal et al., 2018). In this regard, more than half of the world’s population gets survival energy from rice (Rasheed et al., 2020).

Environmental change factors affect the frequency and magnitude of hydrological changes (Turral et al., 2011). It includes biotic and abiotic stresses that are considered a major challenge to agriculture (Turral et al., 2011; Abo Sen et al., 2022). Water deficit is an abiotic factor that induces plant evolution and adversely impacts rice growth and output. Water scarcity is considered a natural phenomenon and is typically characterized as time without significant rainfall. It is the most significant constraint for rice production in rainfed habitats (Nelson et al., 2014; Pandey and Shukla, 2015; Abd El-Aty et al., 2022b). For instance, it causes considerable crop damage and a yield loss of more than 70% compared with normal conditions (Vaghefi et al., 2011; Abd El-Aty et al., 2022c; Abd El-Aty et al., 2022d).

Drought stress induces molecular, physiological, and biochemical alterations depending on genotype, stress severity, and growth stage (Al Azzawi et al., 2020; El-Okkiah et al., 2022a). Furthermore, it stimulates reactive oxygen species (ROS) production, such as O_2_^−^, OH^−^, H_2_O_2_, and O_2_. Overaccumulation of ROS causes oxidative damage and inhibits cell activities. In addition, ROS accumulation leads to chlorophyll breakdown, chloroplast destruction, and a decrease in photosystem II activity (Osakabe et al., 2014). Under stress conditions, to maintain the ROS level, osmolyte (proline) biosynthesis and antioxidant enzyme bioactivity were considerably increased. In recent years, it has become clear that ROS plays a dual role in plants, both as toxic compounds and key regulators of various biological processes such as growth, cell cycle, programmed cell death, hormone signaling, biotic and abiotic cell responses, and development (Mwadzingeni et al., 2016). Many studies indicate that proline protects structural components in cells and the enzymes involved in the antioxidant defense (Naliwajski and Skłodowska, 2021). Therefore, it is important to improve climate-resilient variety development to neutralize the negative effects of environmental stress and preserve sustainable rice production and food security. Improving water deficit tolerance in rice by employing various strategies is challenging due to its complex mechanisms and unpredictable nature (Kumari et al., 2022). These newly developed stress-tolerant rice varieties can mitigate stress-induced plant growth and productivity inhibition (Abid et al., 2018). Overall, developing stress-tolerant rice genotypes is an economically viable and sustainable strategy for enhancing rice production to meet the rising demand for rice (Pandey and Shukla, 2015; Aslam et al., 2022).

In Egypt, the rising population and limited water supply meant that even with increased rice production, the rate of food production lagged behind the demand (Dianga et al., 2020; Abd El-Aty et al., 2022c). Thus, the rice sector’s crop production and yield performance in Egypt has been impressive in the last three decades. The wide adoption of early maturing and semi-dwarf Egyptian varieties expedited a yield of 9.52 t/ha in the 2000s compared with 5.7 t/ha in the 1980s (Abd El-Aty et al., 2022d). Studies of combining ability are one of the many genetic approaches explored to break rice’s yield barrier and increase productivity. It identifies the optimal combiners that can be utilized in crosses to exploit heterosis, collect fixable genes, and obtain desirable segregants. It also aids in comprehending the genetic architecture of diverse traits allowing the breeder to build an effective breeding plan for the future improvement of present materials (Kumari et al., 2022). In order to evaluate different sorts of gene activities with limited means, line × tester mating designs are commonly used. They provide accurate data on the parents’ general and specific combining ability (GCA and SCA) and their cross combinations (Kempthorne, 1957; Dey et al., 2017; Raja et al., 2018).

Improving rice productivity under water deficit by combining conventional breeding methods with modern techniques to obtain water deficit-tolerant rice genotypes is of great economic importance. The present study was conducted to get an idea of the mode of gene action and the magnitude of heterosis, combining the ability for physiological and biochemical traits, yield, and its components.

## 2 Materials and methods

### 2.1 Experimental location

This study was carried out at an experimental farm of the Rice Research and Training Center, Sakha, Kafr Elsheikh, Egypt (31°5′54″N, 30°57′0″E).

According to a previously described method (Page et al., 1983), chemical and mechanical tests of soil and organic matter were conducted at the Agricultural Research Center, Ministry of Agriculture, Egypt. Some chemical and physical parameters of the 0–30-cm deep soil at the experimental site are recorded (**Table S1**). The soil analysis indicated that the soil was clay throughout the profile (56% clay, 32% silt, and 12% sand), with 1.5% organic matter, 8.44 pH, and 3.34 dS/m electrical conductivity.

### 2.2 Rice germplasm used in the study

The experimental material consisted of 10 parents (seven lines: Puebla, Hispagran, IET1444, WAB1573, Giza177, Sakha101, and Sakha105; three testers: Sakha106, Sakha107, and Sakha108), classified into three groups based on their tolerance to drought stress (**Table 1**). In the rice growing season of 2017, the seven female and the three male testers were crossed according to the line × tester mating design to produce 21 F_1_ hybrids as outlined by Kempthorne (1957).

**Table 1:** Origin and main characteristics of the 10 rice genotypes used as parents in the line × tester cross.

No.	Genotype	Parentage	Origin	Variety group	Drought tolerant
Line					
1	Puebla	Unknown	California (USA)	Japonica	Tolerant
2	Hispagran	Unknown	California (USA)	Japonica	Tolerant
3	IET1444	(TN 1 × CO 29)	India	Indica/japonica	Tolerant
4	WAB1573	Introduced	Côte d’Ivoire	Indica	Tolerant
5	Giza177	[Giza171] Ymji No.1//PiNo.4	Egypt	Japonica	Sensitive
6	Sakha101	(Giza176/Milyang 79)	Egypt	Japonica	Moderate
7	Sakha105	GZ5581-46-3/GZ4316-7-1-1	Egypt	Japonica	Sensitive
Testers					
1	Sakha106	(Giza177/Hexi 30)	Egypt	Japonica	Moderate
2	Sakha107	(Giza177/BL1)	Egypt	Japonica	Tolerant
3	Sakha108	(Sakha101/HR5824-B-3-2-3/Sakha101)	Egypt	Japonica	Tolerant

The parents and their F_1_ hybrid seeds were sown in a dry seedbed during the summer season of 2018. Thirty-day-old seedlings of 31 rice genotypes (10 parents and 21 F_1_’s) were individually transplanted in the field plots in two separate irrigation experiments. The first experiment (normal condition) was irrigated every 4 days (14,400 m^3^ ha^−1^), and the plots of this experiment were kept saturated with water from transplanting up to 2 weeks before harvesting. However, the second experiment (water deficit) was irrigated every 10 days (9,120 m^3^ ha^−1^).

As advised by the Ministry of Agriculture, a permanent field was created, and Ca-superphosphate (15.5% P_2_O_5_) was applied at a rate of 238 kg ha^−1^ during soil tillage. At 15 and 35 days after transplanting, two equal doses of potassium sulfate (48% K_2_O) were applied at a rate of 120 kg K_2_O ha^−1^. Nitrogen fertilizers in the form of urea (46% N) at a concentration of 165 unit ha^−1^ of N (357 kg urea ha^−1^) were also applied at 15, 35, and 55 days after transplanting in three equal dosages.

All farming methods were implemented in accordance with the RRTC rice recommendations (RRTC, 2018). Ten plants were selected randomly from each replication to collect data, and the mean values were used for statistical analysis. **Figures S1A, B** display weather data for 2017 and 2018 (rain in mm, the average temperature in °C, relative humidity in %, and radiation in MJ/m^2^) collected from https://power.larc.nasa.gov.

### 2.3 The experiment design

The seedlings of the two experiments were transplanted in a randomized complete block design with three replications. Each replicate contained five rows of parents and three rows of the F_1_ hybrid. The row was 5 m long with a single seedling per hill and a spacing of 20 × 20 cm between rows and hills.

### 2.4 The studied phenotypic traits

#### 2.4.1 Physiological and biochemical traits

Data were collected on flag leaves from randomly selected five plants from each genotype. Samples of leaves and sheaths were collected from 8:00 to 10:00 separately, quickly placed in preweighed zip-sealed bags, and immediately measured to determine the physiological and biochemical traits, viz., relative water content (RWC) using the strategy portrayed by Barrs and Weatherley (1962), fresh weight (FW) of the leaves (immersed in water for 5 h), and turgid weight (TW). Then in an oven at 80°C for 24 h, the samples were dried, and dry weight (DW) was determined. The RWC was calculated as follows: RWC = ((FW − DW)/(TW − DW)) × 100.

Free proline in leaf tissues was determined according to Bates et al. (1973), and leaf samples (0.5 g) were homogenized in 5 ml of sulfosalicylic acid (3%). Almost 2 ml of extract was placed in a tube, and then 2 ml of ninhydrin reagent and 2 ml of glacial acetic acid were included. The reaction mixture was boiled in a water bath at 100°C for 60 min. After cooling the reaction mixture, 6 ml of toluene was added and transferred to a separating funnel. After careful mixing, the chromophore, including toluene, was separated, and absorbance was read at 520 nm in a spectrophotometer against a toluene blank. Proline concentration was recorded utilizing a calibration curve and expressed as mg proline g/FW.

The activity of catalase and peroxidase enzymes was measured as outlined by Aebi (1974), and fresh leaf samples (0.5 g) were homogenized in 5 ml of 50 mM cold K-phosphate buffer (pH 7.8). The homogenates were centrifuged at 10,000×*g* for 20 min at 4°C. The supernatant was utilized to measure the antioxidant enzyme activity as units of mg^−1^ protein. Total chlorophyll content was calculated using the formula of Maxwell and Johnson (2000), and approximately 1 g fresh weight of mixed leaves was homogenized in 5 ml of 85% cold acetone and centrifuged. The extract was diluted to the appropriate volume before the optical density was determined at 663 and 647 nm.

#### 2.4.2 Agronomic and yield traits

Observations were recorded on 25 randomly chosen plants from each genotype. Individual plants were harvested and threshed separately to determine yield traits as recommended by IRRI (1996), viz., days to 50% heading (days; DH), plant height (cm; PH), grain yield plant^−1^ (g; GY), spikelet fertility (%; SF), and 1,000-grain weight (g; GW).

### 2.5 Statistical analysis

The ordinary analysis of RCBD for data on phenotypic traits was done according to Steel and Torrie (1960). Combining ability analysis was performed using the line × tester method according to Kempthorne (1957). The GCA and SCA effects were tested for significance using the least significant difference the least significant difference (LSD) test at 5% and 1%. The GCA : SCA ratio was calculated to investigate the performance effects and assess the relative importance of additive *versus* non-additive gene effects (Singh and Chaudhary, 1977). Heterotic effects were calculated relative to better parents, as outlined by Falconer and Mackay (1996).

Furthermore, appropriate LSD values were calculated to test the significance of heterotic effects according to the formula suggested by Wynne et al. (1970). Heritability includes the broad (h^2^_b_) and narrow sense (h^2^_n_) heritability for the recorded traits estimated according to Griffiths et al. (2000). The additive (σ^2^A) and dominance (σ^2^D) genetic variances were calculated, and the additive and non-additive types of gene actions were performed as described by Verma and Srivastava (2004).

A correlation matrix was constructed using the Pearson coefficient as the studied traits follow the normal distribution. The correlation matrix was calculated using the *GGally* package and the function *ggpairs* in R-Core-Team (2021).

**Figure 1:**
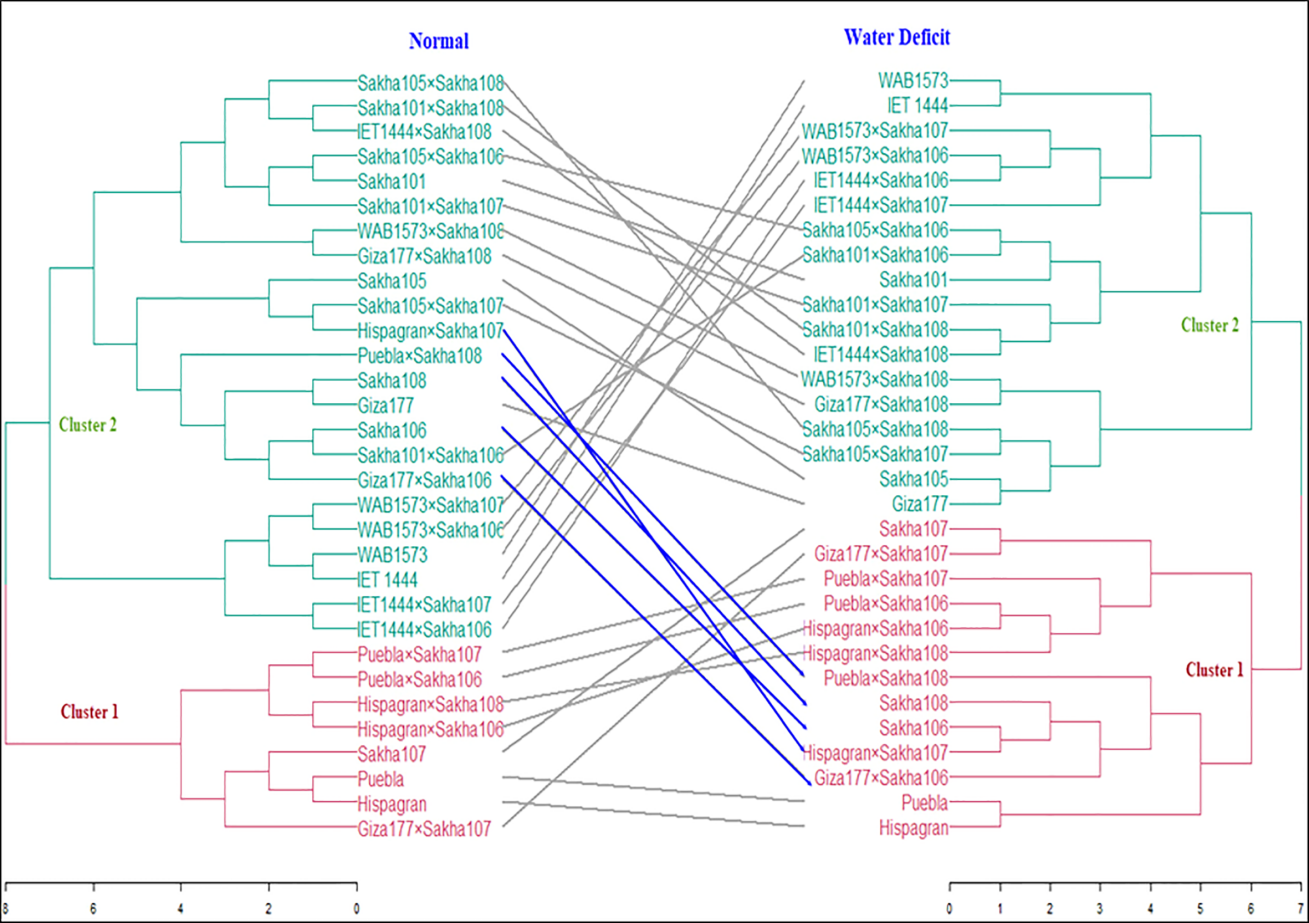
Tanglegram shows cluster analysis results based on the Euclidian coefficient and Ward method under normal and water deficit conditions.

A heatmap was produced using the *pheatmap* package and the function *pheatmap* in R-Core-Team (2021). Data were standardized by subtracting the mean from each value and dividing it by the standard deviation. So all traits will have a mean zero and a standard deviation of 1 and consequently can be compared. The standardization was done due to the different measuring units of the studied traits. After standardization, we can examine the relationship between the traits and the lines. Cluster analysis was performed using R-Core-Team (2021) to classify the genotypes in terms of their drought tolerance by applying the Euclidian distance measure and Ward’s algorithm (Ward, 1963). Under normal and water-deficient conditions, the similarity among all genotypes was demonstrated by constructing a distance matrix and generating a tanglegram based on all examined traits (**Figure 1**). The data were standardized due to their various measurement units. The cubic clustering criterion (CCC) (Milligan and Cooper, 1985) was used to determine whether or not the data contained clusters. Using an agglomerative coefficient, six hard clustering approaches were tested to determine the best precise method for grouping the data. The approaches used were average, generalized average, single, weighted, complete, and the Ward technique. Under normal and water deficit situations, the Ward technique had the largest agglomerative coefficient compared with the other methods; hence, it was chosen to perform the cluster analysis on our data. In addition, internal validation was applied to detect the optimal number of clusters in the data by voting among 30 indices to determine the optimal number of clusters (Charrad et al., 2014).

## 3 Results

### 3.1 Analysis of variance

The mean squares estimates for the ordinary and line × tester analysis for physiological, biochemical, and yield-related traits under regular watering and water deficit conditions are shown in **Table 2**. Highly significant mean squares due to the genotypes and their partitions, parents, crosses, and parent *versus* crosses were detected for all the studied traits except for total chlorophyll content under normal irrigation conditions. The variations among crosses were partitioned into lines, testers, and line × tester. Highly significant differences were detected among partitions for all the studied traits. The comparative estimates of general (σ²GCA) and specific combining ability (σ²SCA) variances showed that the value of the σ²GCA variance was less than the value of the σ²SCA variance. Moreover, the ratio of σ²GCA/σ²SCA was less than unity for all the studied traits.

**Table 2:** Mean square estimates of ordinary, line × tester, and combining ability analyses for physiological, biochemical, and yield-related (and its components) traits under normal watering and water deficit conditions.

Source of variation		*D.F*	RWC (%)		Proline (mg g^−1^ FW)		CAT (unit mg^−1^ protein)		APX (unit mg^−1^ protein)		Total chlorophyll (mg g^−1^ FW)	
			N	W. D	N	W. D	N	W. D	N	W. D	N	W. D
**Genotypes**		30	268.42**	357.50**	0.07**	0.10**	23.36**	18.33**	19.02**	27.08**	1.46**	2.55**
**Parents**		9	289.16**	202.46**	0.02**	0.06**	20.32**	14.03**	7.58**	11.91**	0.42**	1.15**
**Crosses**		20	254.67**	421.26**	0.09**	0.11**	25.74**	18.41**	25.00**	34.92**	2.00**	3.27**
**Parents *vs.* crosses**		1	356.78**	477.68**	0.02*	0.12**	3.05**	55.52**	2.52**	6.79**	0.01	0.55**
**Lines**		6	540.49**	1,098.66**	0.21**	0.24**	42.10**	26.76**	37.47**	66.91**	2.99**	6.54**
**Testers**		2	216.62**	93.64**	0.09**	0.14**	80.85**	63.06**	73.23**	64.72**	6.21**	6.35**
**Lines × testers**		12	118.10**	137.17**	0.03**	0.04**	8.37**	6.79**	10.73**	13.96**	0.80**	1.12**
**Error**		60	2.61	1.71	0.003	0.002	0.04	0.03	0.09	0.07	0.01	0.01
**σ^2^GCA**		–	3.56	7.40	0.002	0.002	0.45	0.30	0.37	0.55	0.03	0.06
**σ^2^SCA**		–	38.50	45.15	0.009	0.014	2.78	2.25	3.55	4.63	0.27	0.37
**σ^2^GCA/σ^2^SCA**		–	0.09	0.16	0.185	0.127	0.16	0.13	0.10	0.12	0.12	0.15
Source of variation		*D.F*	Days to heading (days)		Plant height (cm)		Grain yield/plant (g)		Spikelet fertility (%)		1,000-grain weight (g)	
			N	W. D	N	W. D	N	W. D	N	W. D	N	W. D
**Genotypes**		30	156.56**	143.52**	120.33**	85.80**	131.47**	63.23**	13.99**	23.83**	14.67**	21.17**
**Parents**		9	105.92**	86.53**	74.74**	100.78**	107.78**	42.82**	10.65**	39.50**	13.85**	24.02**
**Crosses**		20	186.39**	175.75**	145.30**	80.82**	122.41**	61.94**	14.90**	16.82**	15.48**	20.63**
**Parents *vs.* crosses**		1	15.45*	11.90**	31.15**	50.73**	525.94**	272.48**	25.62**	22.92**	5.90**	6.29**
**Lines**		6	384.15**	379.42**	327.74**	156.32**	281.80**	146.29**	33.40**	34.80**	36.43**	53.03**
**Testers**		2	228.00**	136.84**	20.58**	85.25**	41.92**	26.49**	5.80**	17.52**	20.48**	14.15**
**Lines × testers**		12	80.58**	80.40**	74.87**	42.32**	56.13**	25.68**	7.17**	7.71**	4.17**	5.51**
**Error**		60	2.27	1.49	2.36	1.64	2.34	1.72	0.35	0.35	0.53	0.45
**σ^2^GCA**		–	2.76	2.48	1.83	1.00	1.73	0.94	0.20	0.24	0.29	0.39
**σ^2^SCA**		–	26.11	26.30	24.17	13.56	17.93	7.99	2.27	2.45	1.21	1.69
**σ^2^GCA/σ^2^SCA**		–	0.11	0.09	0.08	0.07	0.10	0.12	0.09	0.10	0.24	0.23

* and ** indicate significance at 0.05 and 0.01 levels of probability, respectively.

N, normal condition; W. D, water deficit condition.

### 3.2 Mean performance

#### 3.2.1 Physiological and biochemical traits

The mean performance of the studied genotypes (lines, testers, and their F_1_ crosses**)** for yield traits under regular irrigation and water deficit conditions is shown in **Table 3**. The mean performance of the investigated traits varied from cross to cross. Water deficit depressingly impacted the RWC. Hispagran gave the highest values (87.70% and 75.43% under normal and water deficit conditions, respectively) among the lines. The crosses Puebla × Sakha107 and Hispagran × Sakha108 revealed the highest mean values for relative water content compared with other crosses, which recorded 97.70% and 96.60% under normal conditions and 89.87% and 91.69% under water deficit conditions, respectively. Otherwise, water scarcity caused a considerable increase in proline content and the activities of antioxidant enzymes. For proline content, the highest values were recorded by the genotypes Hispagran and Hispagran × Sakha108 (1.60 and 1.77, respectively) under normal conditions and Puebla and Puebla × Sakha106 (2.06 and 2.10, respectively) under water deficit conditions. The maximum activity of CAT was recorded for the genotypes Hispagran, Puebla, Hispagran × Sakha106, and Puebla × Sakha106 (23.52, 23.29, 21.23, 24.20, and 24.00 under normal conditions and 26.13, 25.72, 25.53, 26.92, and 26.75 under drought stress, respectively). Regarding APX, Puebla recorded the highest APX antioxidant enzyme activity compared with other lines, with values of 18.98 and 22.50 under normal and water deficit conditions, respectively. Meanwhile, the highest values were detected in the hybrids Hispagran × Sakha106, Puebla × Sakha106, and Hispagran × Sakha108 under normal irrigation, i.e., 22.14, 20.78, and 20.49, respectively. In addition, Hispagran × Sakha108 and Hispagran × Sakha106 showed 27.09 and 26.18 under water deficit conditions, respectively. Water shortage decreased the total chlorophyll content, where the lines Hispagran and Puebla showed the highest values of total chlorophyll content among parents (8.68 and 8.52 under normal conditions and 8.07 and 7.77 water deficit conditions, respectively). At the same time, the highest values among the F_1_ hybrids were observed in the cross Puebla × Sakha106, followed by Giza177 × Sakha107 (9.43 and 9.35, respectively) under normal conditions and the crosses Puebla × Sakha107 and Hispagran × Sakha106 (8.64 and 8.42, respectively) under water deficit condition.

**Table 3:** Mean performance of lines, testers, and their F_1_ crosses for physiological, biochemical, and yield-related (and its components) traits under normal watering and water deficit conditions.

Genotypes		RWC (%)		Proline (mg g^−1^ FW)		CAT (unit mg^−1^ protein)		APX (unit mg^−1^ protein)		Total chlorophyll (mg g^−1^ FW)	
		N	W. D	N	W. D	N	W. D	N	W. D	N	W. D
**Lines**	**Puebla**	86.20	73.17	1.52	2.06	21.23	25.53	18.98	22.50	8.52	7.77
	**Hispagran**	87.70	75.43	1.60	1.92	23.52	26.13	17.41	21.60	8.68	8.07
	**IET1444**	65.03	61.03	1.49	1.66	20.06	23.93	16.36	19.16	7.59	6.48
	**WAB1573**	62.73	57.46	1.50	1.71	18.76	23.39	17.94	19.30	7.89	6.18
	**Giza177**	76.35	61.83	1.45	1.82	16.16	19.70	14.44	16.31	7.90	6.62
	**Sakha101**	77.28	56.84	1.46	1.67	16.45	22.48	15.40	17.31	7.52	6.98
	**Sakha105**	82.43	73.57	1.31	1.77	17.73	20.33	14.39	18.49	7.85	6.48
**Testers**	**Sakha106**	79.17	62.00	1.50	1.89	19.24	24.11	17.24	19.56	7.97	6.75
	**Sakha107**	95.07	80.17	1.58	1.96	23.29	25.72	18.23	21.86	8.29	7.37
	**Sakha108**	79.10	66.27	1.58	1.97	17.77	23.22	15.86	20.41	8.17	7.43
**Crosses**	**Puebla × Sakha106**	92.07	84.73	1.74	2.10	24.00	26.75	20.78	25.31	9.43	8.33
	**Puebla × Sakha107**	97.70	89.87	1.73	2.07	22.60	22.25	20.02	24.49	9.14	8.64
	**Puebla × Sakha108**	70.07	75.78	1.52	1.90	18.10	19.65	17.00	20.18	7.99	6.43
	**Hispagran × Sakha106**	93.63	86.20	1.76	2.01	24.20	26.92	22.14	26.18	9.03	8.42
	**Hispagran × Sakha107**	90.51	67.64	1.48	1.84	20.10	22.30	14.28	18.97	7.87	6.83
	**Hispagran × Sakha108**	96.60	91.69	1.77	2.03	22.10	24.61	20.49	27.09	8.48	7.78
	**IET1444 × Sakha106**	72.64	64.35	1.42	1.87	17.64	21.80	18.24	20.67	7.89	6.93
	**IET1444 × Sakha107**	80.88	65.72	1.45	1.53	18.94	19.50	17.97	19.90	8.30	7.71
	**IET1444 × Sakha108**	72.90	66.19	1.44	1.62	17.75	20.13	13.78	16.36	7.22	6.43
	**WAB1573 × Sakha106**	75.00	63.20	1.52	1.82	17.31	21.86	19.59	22.15	8.43	6.74
	**WAB1573 × Sakha107**	79.73	64.57	1.46	1.56	19.10	21.23	19.11	22.20	8.45	6.93
	**WAB1573 × Sakha108**	81.00	72.33	1.30	1.50	12.99	17.98	17.96	20.22	6.63	5.95
	**Giza177 × Sakha106**	89.60	77.78	1.37	1.71	19.95	23.40	18.38	20.94	8.18	7.63
	**Giza177 × Sakha107**	96.40	87.03	1.15	1.98	21.72	24.80	14.02	17.82	9.35	8.33
	**Giza177 × Sakha108**	89.89	87.17	1.20	1.61	15.94	20.42	14.57	16.81	7.54	6.05
	**Sakha101 × Sakha106**	79.06	60.25	1.60	1.79	20.28	22.40	17.31	19.69	8.30	6.03
	**Sakha101 × Sakha107**	75.04	54.87	1.44	1.65	19.74	22.19	12.33	16.03	7.87	6.35
	**Sakha101 × Sakha108**	68.19	62.97	1.37	1.74	16.45	20.08	14.31	16.24	6.92	5.48
	**Sakha105 × Sakha106**	79.85	55.00	1.41	1.71	19.10	21.90	17.50	20.83	7.28	5.55
	**Sakha105 × Sakha107**	85.86	64.53	1.45	1.56	17.52	19.79	12.92	16.05	7.13	5.82
	**Sakha105 × Sakha108**	82.57	62.23	1.31	1.52	14.10	17.92	13.89	16.73	6.96	5.39
**LSD 0.05**		2.64	2.13	0.10	0.07	0.31	0.30	0.48	0.43	0.12	0.12
**LSD 0.01**		3.51	2.84	0.13	0.09	0.42	0.40	0.64	0.57	0.16	0.16
Genotypes		Days to heading (days)		Plant height (cm)		Grain yield/plant (g)		Spikelet fertility (%)		1,000-grain weight (g)	
		N	W. D	N	W. D	N	W. D	N	W. D	N	W. D
**Lines**	**Puebla**	94.90	89.00	112.17	96.67	49.35	33.81	95.53	89.83	33.08	32.81
	**Hispagran**	95.90	90.00	110.80	100.90	50.89	35.42	95.40	88.92	32.44	30.80
	**IET1444**	103.90	97.00	118.13	108.67	39.13	34.80	91.31	85.31	27.23	24.97
	**WAB1573**	109.67	103.00	117.20	109.53	31.83	28.47	90.83	82.38	27.83	23.40
	**Giza177**	98.90	90.00	114.33	109.17	46.83	31.23	94.43	88.39	29.60	27.69
	**Sakha101**	104.90	94.00	106.00	99.67	46.43	38.93	92.73	80.88	27.04	26.16
	**Sakha105**	93.90	88.00	107.17	96.40	42.32	30.28	93.14	89.09	29.96	26.38
**Testers**	**Sakha106**	95.90	89.00	109.17	98.20	43.33	36.13	94.60	87.70	30.88	28.64
	**Sakha107**	89.90	84.00	102.17	94.17	48.78	38.23	95.52	91.71	28.70	27.55
	**Sakha108**	99.90	94.00	112.17	98.17	50.93	39.63	96.28	91.39	31.63	30.06
**Crosses**	**Puebla × Sakha106**	85.57	80.83	105.48	90.65	56.53	42.73	95.83	88.94	34.65	32.95
	**Puebla × Sakha107**	84.90	78.83	101.04	86.70	60.06	47.24	95.25	90.59	32.94	31.99
	**Puebla × Sakha108**	96.90	89.83	116.71	98.57	53.49	41.64	91.23	87.89	32.40	31.42
	**Hispagran × Sakha106**	91.90	79.83	101.40	94.69	55.79	40.48	96.35	90.66	34.30	32.87
	**Hispagran × Sakha107**	94.90	87.83	111.23	103.72	52.03	40.59	93.51	89.78	30.49	28.91
	**Hispagran × Sakha108**	91.57	83.83	101.91	93.86	56.36	43.78	95.76	94.06	33.80	32.01
	**IET1444 × Sakha106**	98.57	94.83	127.67	99.65	43.53	34.17	88.30	86.00	29.60	27.00
	**IET1444 × Sakha107**	102.23	99.13	124.47	97.14	34.73	29.70	91.00	86.33	26.50	23.00
	**IET1444 × Sakha108**	96.90	93.77	119.57	102.90	50.43	37.17	89.33	88.39	29.10	27.67
	**WAB1573 × Sakha106**	103.33	96.83	114.67	106.26	37.63	33.17	92.90	88.00	29.30	27.00
	**WAB1573 × Sakha107**	110.23	98.83	114.47	103.42	47.03	37.17	91.96	88.57	26.40	24.67
	**WAB1573 × Sakha108**	116.90	108.83	113.63	105.47	45.13	35.17	92.09	88.02	31.00	27.00
	**Giza177 × Sakha106**	95.90	90.83	114.08	99.23	49.79	34.68	92.66	89.63	30.65	28.74
	**Giza177 × Sakha107**	90.23	79.83	103.69	93.58	57.56	43.46	96.65	93.31	31.06	28.68
	**Giza177 × Sakha108**	108.90	97.83	113.67	103.82	54.27	38.89	94.34	89.67	30.65	28.33
	**Sakha101 × Sakha106**	96.90	90.83	112.10	103.09	47.77	38.04	92.85	85.60	29.36	27.11
	**Sakha101 × Sakha107**	99.57	94.83	108.82	100.99	50.19	42.61	91.64	86.54	27.76	26.21
	**Sakha101 × Sakha108**	98.90	92.83	113.73	103.09	52.52	42.22	92.24	87.32	29.35	28.06
	**Sakha105 × Sakha106**	99.90	94.83	110.69	101.17	46.80	36.37	91.84	84.13	28.89	26.17
	**Sakha105 × Sakha107**	89.90	85.83	114.13	98.65	48.33	33.86	93.23	89.00	29.50	28.73
	**Sakha105 × Sakha108**	101.90	90.83	112.40	104.41	45.24	32.39	91.00	88.61	30.19	27.93
	**LSD 0.05**	2.46	1.99	2.51	2.09	2.50	2.14	0.97	0.97	1.19	1.10
	**LSD 0.01**	3.27	2.65	3.34	2.78	3.32	2.85	1.29	1.29	1.58	1.46

N, normal condition; W. D, water deficit condition.

#### 3.2.2 Yield traits

For days to heading, compared with other parental lines, the earliest parents in heading date were Sakha105, followed by Puebla and Hispagran under both irrigation treatments as they recorded the lowest desirable values of 93.9, 94.9, and 95.9 days under normal irrigation and 88, 89, and 90 days under water deficit condition, respectively. On the other hand, the two parents, WAB1573 and IET1444, were the latest parents in heading date under normal and water deficit conditions and recorded the highest values of 109.67 and 103.90 days under regular irrigation and 103 and 97 days under water shortage conditions, respectively. The best hybrid combinations that gave the lowest desirable mean values for heading date were Puebla × Sakha107 and Puebla × Sakha106 under normal conditions. Moreover, Puebla × Sakha107, Hispagran × Sakha106, and Giza177 × Sakha107 under water deficit conditions showed 84.90, 85.57, 78.83, 79.83, and 79.83 days to 50% flowering, respectively.

For plant height, the results showed that the shortest and desirable lines were Sakha101 and Sakha105 under normal irrigation, which recorded values of 106.00 and 107.17 cm, respectively, and Sakha105 and Puebla under water deficit conditions 96.40 and 96.67 cm, respectively. These lines performed well for plant height under both conditions. Among the F_1_ hybrids, the combination Puebla × Sakha107 recorded the lowest desirable plant height values under normal and water deficit conditions (101 and 86.70 cm, respectively), followed by Hispagran × Sakha106 and Hispagran × Sakha108 under normal irrigation (101 and 101 cm) and Puebla × Sakha106 under water shortage condition (90.65 cm).

The highest mean values for single plant yield were detected by the parent lines Hispagran (50.89 g) and Puebla (49.35 g) under normal irrigation. In contrast, the parent Sakha101 showed the highest grain yield/plant under water deficit conditions (38.93 g). Among the 21 crosses, hybrid combinations Puebla × Sakha107 and Giza177 × Sakha107 were the best compared with the other crosses. They recorded the highest grain yield/plant under both irrigation treatments with values of 60.06 and 57.56 g under normal irrigation and 47.24 and 43.46 g under water deficit conditions, respectively. However, the cross IET1444 × Sakha107 recorded the lowest mean values of grain yield (34.73 and 29.70 g) under normal and water shortage conditions, respectively.

Data revealed that the parents Puebla and Hispagran and the crosses Giza177 × Sakha107 and Hispagran × Sakha106 gave the highest fertility percentage under the normal condition with values of 95.53% and 95.40% for parents and 96.65% and 96.35% for the crosses, respectively. The parents Puebla and Sakha105, in addition to the crosses Hispagran × Sakha108 and Giza177 × Sakha107, recorded the highest mean performance values for fertility percentage with values of 89.83%, 89.09%, 94.06%, and 93.31%, respectively, under water deficit condition.

Concerning the 1,000-grain weight, results showed that the parental lines Puebla and Hispagran had the heaviest 1,000-grain weight among the parents under both irrigation conditions; their weights were 33.08 and 32.44 g under normal conditions and 32.81 and 30.80 g under water deficit condition, respectively. In F_1_ hybrids, the highest 1,000-grain weight under normal and water deficit conditions (34.65 and 32.95 g, respectively) was observed in the cross Puebla × Sakha106, followed by Hispagran × Sakha106, which recorded 34.30 and 32.87 g under normal and water deficit conditions, respectively. In contrast, the lowest 1,000-grain weights under normal irrigation (26.40 and 27.04 g) were observed in the cross WAB1573 × Sakha107 and the parent Sakha101, respectively. In comparison, the hybrid IET1444 × Sakha107 and the parent WAB1573 exhibited minimum values under water deficit conditions (23.00 and 23.40 g, respectively).

### 3.3 General combining abilities effect

#### 3.3.1 Physiological and biochemical traits

The estimates of the individual parent’s GCA effects for each trait were recorded (**Table 4**). The positive GCA estimates are pivotal for all physiological and biochemical traits. Data illustrated that the parental lines Puebla, Hispagran, and Giza177 showed significant and desirable GCA effects of RWC and CAT activity under both conditions. Moreover, the lines Puebla and Hispagran were the best general combiners for the proline content under both irrigation conditions, as they recorded the maximum significant and positive GCA effects for that trait. Meanwhile, Puebla, Hispagran, and WAB1573, under both irrigation treatments, were the best combiners for APX activity, expressing the highest favorable and significant GCA estimates for APX activity. Moreover, significant and desirable GCA effects of total chlorophyll content were detected by IET1444 under water deficit conditions and by Puebla, Hispagran, and Giza177 under both conditions for total chlorophyll content.

**Table 4:** Estimates of general combining ability (GCA) effects of the parents for physiological, biochemical, and yield-related (and its components) traits under normal watering and water deficit conditions.

	RWC (%)			Proline (mg g^−1^ FW)		CAT (unit mg^−1^ protein)		APX (unit mg^−1^ protein)		Total chlorophyll (mg g^−1^ FW)	
Line	N	W. D		N	W. D	N	W. D	N	W. D	N	W. D
Puebla	3.32**	11.84**		0.19**	0.26**	2.54**	1.08**	2.29**	3.10**	0.84**	0.96**
Hispagran	10.29**	10.22**		0.20**	0.19**	3.10**	2.81**	1.99**	3.85**	0.44**	0.83**
IET1444	−7.82**	−6.21**		−0.04	−0.09**	−0.92**	−1.33**	−0.32*	−1.25**	−0.21**	0.18**
WAB1573	−4.72**	−4.92**		−0.05	−0.14**	−2.56**	−1.45**	1.90**	1.29**	−0.18**	−0.30**
Giza177	8.67**	12.37**		−0.23**	0.00	0.17*	1.07**	−1.32**	−1.71**	0.34**	0.49**
Sakha101	−9.20**	−12.26**		0.00	−0.04*	−0.21*	−0.25**	−2.33**	−2.91**	−0.32**	−0.90**
Sakha105	−0.54	−11.04**		−0.08**	−0.17**	−2.12**	−1.93**	−2.21**	−2.36**	−0.90**	−1.26**
LSD (GI) 0.05	1.32	1.07		0.05	0.03	0.16	0.15	0.24	0.21	0.06	0.06
LSD (GI) 0.01	2.00	1.61		0.07	0.05	0.24	0.23	0.36	0.32	0.09	0.09
Testers											
Sakha106	−0.17	−1.41*		0.07*	0.09**	1.32**	1.77**	2.15**	2.02**	0.34**	0.24**
Sakha107	3.30*	−1.02		−0.02	−0.03	0.93**	−0.08	−1.17**	−0.88**	0.28**	0.38**
Sakha108	−3.12*	2.43*		−0.05	−0.07*	−2.25**	−1.69**	−0.98**	−1.14**	−0.63**	−0.63**
LSD (GI) 0.05	1.52	1.23		0.06	0.04	0.18	0.17	0.28	0.25	0.07	0.07
LSD (GI) 0.01	3.50	2.83		0.13	0.09	0.42	0.40	0.64	0.57	0.16	0.16
	Days to heading (days)			Plant height (cm)		Grain yield/plant (g)		Spikelet fertility (%)		1,000-grain weight (g)	
Line	N		W. D	N	W. D	N	W. D	N	W. D	N	W. D
Puebla	−8.78**		−7.87**	−4.43**	−7.60**	6.92**	5.51**	1.25**	0.52*	2.95**	3.72**
Hispagran	−5.12**		−7.20**	−7.32**	−2.15**	4.95**	3.26**	2.35**	2.88**	2.49**	2.86**
IET1444	1.33*		4.88**	11.73**	0.32	−6.87**	−4.68**	−3.31**	−1.71**	−1.98**	−2.51**
WAB1573	12.25**		10.47**	2.09**	5.48**	−6.51**	−3.19**	−0.54*	−0.42	−1.48**	−2.18**
Giza177	0.44		−1.53**	−1.69*	−0.70	4.10**	0.65	1.70**	2.25**	0.41	0.18
Sakha101	0.55		1.80**	−0.62	2.81**	0.39	2.60**	−0.61*	−2.14**	−1.55**	−1.28**
Sakha105	−0.67		−0.53	0.24	1.84**	−2.99**	−4.15**	−0.83**	−1.37**	−0.85*	−0.79*
LSD (GI) 0.05	1.23		1.00	1.25	1.04	1.25	1.07	0.48	0.48	0.59	0.55
LSD (GI) 0.01	1.86		1.51	1.90	1.58	1.89	1.62	0.73	0.73	0.90	0.83
Testers											
Sakha106	−1.90*		−1.20*	0.13	−0.33	−1.51*	−1.27*	0.11	−1.05*	0.59	0.43
LSD (GI) 0.05	1.41		1.15	1.44	1.20	1.44	1.23	0.56	0.56	0.68	0.63
Sakha108	3.80**		2.93**	0.92	2.16*	1.29	0.39	−0.57*	0.51	0.55	0.51
LSD (GI) 0.05	1.41		1.15	1.44	1.20	1.44	1.23	0.56	0.56	0.68	0.63
LSD (GI) 0.01	3.26		2.64	3.33	2.77	3.32	2.84	1.29	1.28	1.57	1.45

* and ** indicate significance at 0.05 and 0.01 levels of probability, respectively.

N, normal condition; W. D, water deficit condition.

#### 3.3.2 Yield traits

As shown, the lines Puebla and Hispagran gave the highest significant negative and desirable GCA effects for earliness and short stature. In addition, they gave the highest positive significant values for grain yield/plant and 1,000-grain weight under both irrigation conditions. In contrast, the parent Hispagran followed by Giza177 showed the highest significant GCA effects for spikelet fertility percentage under normal and water deficit conditions. In contrast, the two parents IET1444 and WAB1573 expressed the highest significant undesirable GCA effects for all the yield traits under normal and water deficit conditions.

### 3.4 Specific combining abilities effect

#### 3.4.1 Physiological and biochemical traits

The estimates of specific combining ability (SCA) effects of all combinations for all the studied traits were recorded (**Table 5**). Out of the 21 crosses, two crosses under normal conditions, three crosses under water deficit conditions, and five crosses under both conditions showed significant positive SCA effects for RWC, while Puebla × Sakha107, followed by Hispagran × Sakha108, recorded the highest SCA estimates for RWC under both irrigation conditions. For proline content, high SCA effects were recorded for Sakha105 × Sakha107 under normal irrigation, IET1444 × Sakha106, WAB1573 × Sakha106, Giza177 × Sakha107, and Sakha101 × Sakha108 under water deficit conditions, and Hispagran × Sakha108 and Puebla × Sakha107 under both conditions. In the case of the activities of antioxidant enzymes, one cross combination under water deficit condition and seven cross combinations under both conditions were the best specific combiners. They showed positive and significant SCA effects for CAT; among them, Hispagran × Sakha108 and Puebla × Sakha106 gave the highest significant SCA values for CAT antioxidant enzyme under normal and water deficit conditions, respectively.

**Table 5:** Estimates of specific combining ability (SCA) effects of the F_1_ hybrids for physiological, biochemical, and yield-related (and its components) traits under normal watering and water deficit conditions.

Genotypes	RWC (%)		Proline (mg g^−1^ FW)			CAT (unit mg^−1^ protein)		APX (unit mg^−1^ protein)				total chlorophyll (mg g^−1^ FW)		
	N	W. D	N	W. D		N	W. D	N		W. D		N	W. D	
**Puebla × Sakha106**	5.63**	2.68**	0.00	−0.01		1.11**	2.09**	−0.64**		−0.04		0.23**	0.29**	
**Puebla × Sakha107**	7.79**	7.43**	0.09*	0.07**		0.10	−0.55**	1.93**		2.04**		0.00	0.46**	
**Puebla × Sakha108**	−13.42**	−10.11**	−0.09*	−0.06*		−1.21**	−1.54**	−1.29**		−2.01**		−0.24**	−0.74**	
**Hispagran × Sakha106**	0.22	5.77**	0.01	−0.04		0.74**	0.54**	1.02**		0.08		0.23**	0.50**	
**Hispagran × Sakha107**	−6.36**	−13.18**	−0.17**	−0.09**		−2.96**	−2.23**	−3.52**		−4.23**		−0.87**	−1.23**	
**Hispagran × Sakha108**	6.14**	7.42**	0.16**	0.14**		2.22**	1.69**	2.50**		4.15**		0.65**	0.73**	
**IET1444 × Sakha106**	−2.66**	0.34	−0.09*	0.11**		−1.79**	−0.45**	−0.58**		−0.33*		−0.26**	−0.34**	
**IET1444 × Sakha107**	2.11*	1.32	0.03	−0.12**		−0.10	−0.90**	2.48**		1.80**		0.21**	0.30**	
**IET1444 × Sakha108**	0.54	−1.66*	0.06	0.01		1.89**	1.34**	−1.90**		−1.48**		0.04	0.04	
**WAB1573 × Sakha106**	−3.40**	−2.10*	0.02	0.10**		−0.48**	−0.27*	−1.45**		−1.39**		0.25**	−0.04	
**WAB1573 × Sakha107**	−2.14*	−1.11	0.05	−0.04		1.70**	0.95**	1.40**		1.56**		0.33**	0.00	
**WAB1573 × Sakha108**	5.54**	3.21**	−0.07*	−0.06*		−1.22**	−0.69**	0.06		−0.16		−0.58**	0.04	
**Giza177 × Sakha106**	−2.19*	−4.81**	0.06	−0.15**		−0.58**	−1.25**	0.57**		0.40*		−0.52**	0.05	
**Giza177 × Sakha107**	1.14	4.06**	−0.07*	0.24**		1.59**	2.01**	−0.46*		0.18		0.71**	0.61**	
**Giza177 × Sakha108**	1.05	0.75	0.02	−0.09**		−1.01**	−0.76**	−0.11		−0.57**		−0.19**	−0.66**	
**Sakha101 × Sakha106**	5.13**	2.30**	0.05	−0.03		0.13	−0.93**	0.51**		0.35*		0.26**	−0.16**	
**Sakha101 × Sakha107**	−2.35*	−3.47**	−0.01	−0.05*		−0.01	0.71**	−1.15**		−0.41*		−0.11*	0.01	
**Sakha101 × Sakha108**	−2.78**	1.18	−0.04	0.08**		−0.12	0.21	0.64**		0.06		−0.15**	0.15**	
**Sakha105 × Sakha106**	−2.74**	−4.18**	−0.05	0.02		0.87**	0.26*	0.58**		0.94**		−0.19**	−0.29**	
**Sakha105 × Sakha107**	−0.19	4.96**	0.08*	−0.01		−0.32**	0.00	−0.68**		−0.94**		−0.27**	−0.15**	
**Sakha105 × Sakha108**	2.93**	−0.79	−0.03	−0.01		−0.55**	−0.26*	0.10		0.00		0.47**	0.43**	
**LSD 0.05**	1.95	1.57	0.07	0.05		0.23	0.22	0.35		0.32		0.09	0.09	
**LSD 0.01**	2.65	2.15	0.10	0.07		0.32	0.30	0.48		0.43		0.12	0.12	
Genotypes	Days to heading (days)		Plant height (cm)			Grain yield/plant (g)		Spikelet fertility (%)				1,000-grain weight (g)		
	N	W. D	N		W. D	N	W. D	N		W. D		N		W. D	
**Puebla × Sakha106**	−1.66	−1.13	−2.39*		−1.00	1.35	0.13	1.62**		0.86*		0.73		0.40	
**Puebla × Sakha107**	−2.31*	−2.60**	−5.66**		−3.44**	3.15**	2.49**	0.68		0.91*		0.75		0.81*	
**Puebla × Sakha108**	3.97**	3.73**	8.05**		4.44**	−4.50**	−2.62**	−2.30**		−1.76**		−1.48**		−1.21**	
**Hispagran × Sakha106**	1.01	−2.80**	−3.58**		−2.41**	2.57**	0.13	1.04**		0.22		0.85		1.17**	
**Hispagran × Sakha107**	4.02**	5.73**	7.43**		8.13**	−2.91**	−1.90*	−2.16**		−2.26**		−1.23**		−1.41**	
**Hispagran × Sakha108**	−5.03**	−2.93**	−3.86**		−5.72**	0.34	1.77*	1.12**		2.04**		0.39		0.23	
**IET1444 × Sakha106**	1.23	0.12	3.64**		0.08	2.14*	1.76*	−1.35**		0.15		0.61		0.68	
**IET1444 × Sakha107**	4.91**	4.95**	1.62		−0.93	−8.38**	−4.85**	0.99**		−1.11**		−0.76		−1.94**	
**IET1444 × Sakha108**	−6.14**	−5.08**	−5.25**		0.85	6.24**	3.10**	0.36		0.97*		0.15		1.26**	
**WAB1573 × Sakha106**	−4.93**	−3.47**	0.28		1.54	−4.12**	−0.73	0.48		0.86*		−0.19		0.34	
**WAB1573 × Sakha107**	1.99*	−0.94	1.26		0.20	3.55**	1.13	−0.82*		−0.17		−1.36**		−0.61	
**WAB1573 × Sakha108**	2.94**	4.40**	−1.54		−1.74*	0.58	−0.39	0.34		−0.69		1.55**		0.26	
**Giza177 × Sakha106**	−0.55	2.53**	3.47**		0.68	−2.58**	−3.06**	−1.99**		−0.19		−0.72		−0.28	
**Giza177 × Sakha107**	−6.20**	−7.94**	−5.74**		−3.47**	3.47**	3.58**	1.63**		1.90**		1.41**		1.05*	
**Giza177 × Sakha108**	6.75**	5.40**	2.27*		2.78**	−0.89	−0.51	0.36		−1.72**		−0.69		−0.77	
**Sakha101 × Sakha106**	0.34	−0.80	0.42		1.03	−0.88	−1.65*	0.50		0.17		−0.05		−0.45	
**Sakha101 × Sakha107**	3.02**	3.73**	−1.68		0.43	−0.19	0.78	−1.07**		−0.49		0.08		0.03	
**Sakha101 × Sakha108**	−3.36**	−2.93**	1.26		−1.46	1.07	0.87	0.56		0.32		−0.02		0.42	
**Sakha105 × Sakha106**	4.56**	5.53**	−1.85		0.08	1.52	3.43**	−0.29		−2.06**		−1.22**		−1.87**	
**Sakha105 × Sakha107**	−5.42**	−2.94**	2.77**		−0.93	1.32	−1.22	0.75*		1.22**		1.11*		2.07**	
**Sakha105 × Sakha108**	0.86	−2.60**	−0.93		0.85	−2.84**	−2.21**	−0.45		0.84*		0.11		−0.20	
**LSD 0.05**	1.81	1.47	1.85		1.54	1.84	1.58	0.71		0.71		0.73		0.81	
**LSD 0.01**	2.47	2.01	2.52		2.10	2.52	2.15	0.98		0.97		0.75		1.10	

* and ** indicate significance at 0.05 and 0.01 levels of probability, respectively.

N, normal condition; W. D, water deficit condition.

On the other hand, three hybrid combinations under normal irrigation and seven hybrid combinations under water shortage conditions revealed either significant or highly significant positive SCA effects on APX activity. Among them, Hispagran × Sakha108 was the best specific combiner under both irrigation treatments, followed by IET1444 × Sakha107 under normal conditions and Puebla × Sakha107 under water deficit conditions; this gave the highest desirable SCA values for APX enzyme activity. Regarding total chlorophyll content, the SCA effects were positive and significant for three hybrids under normal conditions. Regarding total chlorophyll content, the SCA effects were positive and significant for three hybrids under normal conditions, two hybrids under water scarcity conditions and six hybrids under both conditions. The highest desirable effects for total chlorophyll content were recorded for Giza177 × Sakha107 and Hispagran × Sakha108 under normal irrigation and water shortage conditions, respectively.

#### 3.4.2 Yield traits

For days to heading, seven crosses out of 21 cross combinations under normal conditions and 10 hybrid combinations under water deficit conditions possessed significant negative SCA effects for days to heading (**Table 6**). On the other hand, the highest negative significant estimates were observed in the hybrid combinations Giza177 × Sakha107 and IET1444 × Sakha108 under both conditions. Regarding plant height, six crosses under normal conditions and four hybrids under water deficit conditions exhibited either significant or highly significant negative SCA effects. The four combinations Giza177 × Sakha107, Hispagran × Sakha108, Puebla × Sakha107, and IET1444 × Sakha108 expressed the highest significant negative SCA effects toward shortness under both conditions. The SCA estimates of grain yield/plant were either significant or highly significant and positive for six crosses under normal and water deficit conditions. The crosses IET1444 × Sakha108 under normal irrigation and Giza177 × Sakha107 followed by Sakha105 × Sakha106 under water deficit conditions exhibited the highest positive (desirable) estimates of SCA effects. The crosses Giza177 × Sakha107 and Puebla × Sakha106 were the best specific combiners under normal conditions that recorded the highest significant SCA effects for fertility percentage among the six crosses that gave desirable and significant effects for spikelet fertility percentage. On the other hand, under water shortage conditions, eight crosses showed either significant or highly significant positive SCA estimates for fertility percentage. Among them, the crosses Hispagran × Sakha108 and Giza177 × Sakha107 were the best specific combiners with the highest positive values of SCA estimates compared with the other eight crosses. The best specific combiners for the 1,000-grain weight were the crosses WAB1573 × Sakha108 and Giza177 × Sakha107 under normal conditions and Sakha105 × Sakha107 under water deficit conditions, which showed the highest significant positive values of SCA effects for the 1,000-grain weight.

**Table 6:** Estimates of heterosis as a deviation from a better parent (heterobeltiosis) of the F_1_ hybrids for physiological, biochemical, and yield-related (and its components) traits under normal watering and water deficit conditions.

Genotypes	RWC (%)		Proline (mg g^−1^ FW)		CAT (unit mg^−1^ protein)		APX (unit mg^−1^ protein)		Total chlorophyll (mg g^−1^ FW)		
	N	W. D	N	W. D	N	W. D	N	W. D	N		W. D
**Puebla × Sakha106**	6.81**	15.80**	14.00**	1.78	13.07**	4.79**	9.48**	12.47**	10.66**		7.30**
**Puebla × Sakha107**	2.77	12.10**	9.70**	0.32	−2.95**	−13.50**	5.48**	8.83**	7.26**		11.29**
**Puebla × Sakha108**	−18.71**	3.57*	−3.59	−7.93**	−14.73**	−23.02**	−10.43**	−10.33**	−6.23**		−17.17**
**Hispagran × Sakha106**	6.76**	14.27**	9.79**	4.51*	2.91**	3.01**	27.14**	21.19**	4.09**		4.42**
**Hispagran × Sakha107**	−4.79**	−15.63**	−7.71*	−6.29**	−14.53**	−14.67**	−21.68**	−13.24**	−9.29**		−15.29**
**Hispagran × Sakha108**	10.15**	21.55**	10.83**	2.88	−6.02**	−5.83**	17.67**	25.40**	−2.25**		−3.51**
**IET1444 × Sakha106**	−8.24**	3.79*	−5.76	−1.23	−12.05**	−9.59**	5.75**	5.66**	−0.99		2.72**
**IET1444 × Sakha107**	−14.92**	−18.02**	−8.54**	−22.11**	−18.67**	−24.19**	−1.47	−8.98**	0.18		4.71**
**IET1444 × Sakha108**	−7.84**	−0.12	−8.67**	−17.94**	−11.50**	−15.89**	−15.79**	−19.86**	−11.55**		−13.38**
**WAB1573 × Sakha106**	−5.26**	1.93	0.78	−3.88*	−10.02**	−9.34**	9.19**	13.22**	5.78**		−0.09
**WAB1573 × Sakha107**	−16.13**	−19.46**	−7.59*	−20.58**	−17.98**	−17.47**	4.78**	1.54	1.99**		−5.88**
**WAB1573 × Sakha108**	2.41	9.15**	−17.55**	−24.03**	−30.74**	−23.14**	0.13	−0.95	−18.78**		−19.82**
**Giza177 × Sakha106**	13.18**	25.46**	−8.87**	−9.70**	3.71**	−2.96**	6.59**	7.04**	2.64**		13.09**
**Giza177 × Sakha107**	1.40	8.57**	−27.43**	0.85	−6.73**	−3.59**	−23.11**	−18.50**	12.87**		13.12**
**Giza177 × Sakha108**	13.64**	31.54**	−23.89**	−18.44**	−10.28**	−12.07**	−8.15**	−17.65**	−7.72**		−18.52**
**Sakha101 × Sakha106**	−0.14	−2.82	6.21	−5.47**	5.42**	−7.11**	0.39	0.65	4.08**		−13.52**
**Sakha101 × Sakha107**	−21.07**	−31.56**	−8.86**	−15.99**	−15.23**	−13.74**	−32.38**	−26.68**	−5.08**		−13.86**
**Sakha101 × Sakha108**	−13.79**	−4.98**	−12.90**	−11.84**	−7.41**	−13.54**	−9.79**	−20.45**	−15.22**		−26.26**
**Sakha105 × Sakha106**	−3.13	−25.24**	−5.99	−9.70**	−0.71	−9.18**	1.49	6.48**	−8.71**		−17.81**
**Sakha105 × Sakha107**	−9.68**	−19.50**	−8.02*	−20.24**	−24.76**	−23.07**	−29.14**	−26.59**	−13.92**		−20.94**
**Sakha105 × Sakha108**	0.16	−15.41**	−17.12**	−23.01**	−20.64**	−22.84**	−12.44**	−18.05**	−14.73**		−27.38**
Genotypes	Days to heading (days)		Plant height (cm)		Grain yield/plant (g)		Spikelet fertility (%)		1,000-grain weight (g)		
	N	W. D	N	W. D	N	W. D	N	W. D	N	W. D	
**Puebla × Sakha106**	−9.83**	−9.18**	−3.38**	−6.22**	14.54**	18.27**	0.31	−0.99	4.74*	0.44	
**Puebla × Sakha107**	−5.56**	−6.15**	−1.11	−7.92**	21.69**	23.54**	−0.30	−1.23*	−0.42	−2.51	
**Puebla × Sakha108**	2.11	0.94	4.05**	1.97	5.01*	5.07	−5.25**	−3.83**	−2.03	−4.23*	
**Hispagran × Sakha106**	−4.17**	−10.30**	−7.11**	−3.58**	9.62**	12.03**	1.00	1.97**	5.74**	6.71**	
**Hispagran × Sakha107**	5.56**	4.56**	8.87**	10.14**	2.23	6.15*	−2.11**	−2.10**	−6.00**	−6.15**	
**Hispagran × Sakha108**	−4.52**	−6.85**	−8.03**	−4.39**	10.66**	10.48**	−0.54	2.92**	4.21*	3.92*	
**IET1444 × Sakha106**	2.78*	6.55**	16.95**	1.48	0.46	−5.44	−6.66**	−1.93**	−4.13*	−5.74**	
**IET1444 × Sakha107**	13.72**	18.02**	21.83**	3.15**	−28.80**	−22.32**	−4.73**	−5.87**	−7.67**	−16.52**	
**IET1444 × Sakha108**	−3.00*	−0.25	6.60**	4.82**	−0.98	−6.22*	−7.22**	−3.28**	−7.99**	−7.95**	
**WAB1573 × Sakha106**	7.75**	8.80**	5.04**	8.21**	−13.15**	−8.21**	−1.79**	0.35	−5.11*	−5.74**	
**WAB1573 × Sakha107**	22.62**	17.66**	12.04**	9.82**	−3.59	−2.79	−3.72**	−3.43**	−8.01**	−10.47**	
**WAB1573 × Sakha108**	17.02**	15.78**	1.31	7.44**	−11.39**	−11.27**	−4.35**	−3.68**	−1.98	−10.17**	
**Giza177 × Sakha106**	0.00	2.06	4.50**	1.05	13.59**	−4.02	−2.05**	1.40*	−0.73	0.33	
**Giza177 × Sakha107**	0.37	−4.96**	1.49	−0.63	18.00**	13.66**	1.18*	1.75**	4.94*	3.58	
**Giza177 × Sakha108**	10.11**	8.70**	1.34	5.76**	6.55**	−1.88	−2.02**	−1.88**	−3.10	−5.74**	
**Sakha101 × Sakha106**	1.04	2.06	5.76**	4.98**	2.89	−2.31	−1.85**	−2.39**	−4.91*	−5.34**	
**Sakha101 × Sakha107**	10.75**	12.90**	6.51**	7.24**	2.88	9.45**	−4.06**	−5.64**	−3.26	−4.86*	
**Sakha101 × Sakha108**	−1.00	−1.24	7.29**	5.01**	3.12	6.52*	−4.20**	−4.46**	−7.19**	−6.65**	
**Sakha105 × Sakha106**	6.39**	7.77**	3.29**	4.94**	7.99**	0.64	−2.92**	−5.56**	−6.42**	−8.63**	
**Sakha105 × Sakha107**	0.00	2.18	11.71**	4.76**	−0.93	−11.45**	−2.40**	−2.95**	−1.52	4.30*	
**Sakha105 × Sakha108**	8.52**	3.22**	4.88**	8.31**	−11.19**	−18.28**	−5.49**	−3.04**	−4.55*	−7.09**	

* and ** indicate significance at 0.05 and 0.01 levels of probability, respectively.

N, normal condition; W. D, water deficit condition.

### 3.5 Estimates of heterosis as a deviation from the better parent (heterobeltiosis)

#### 3.5.1 Physiological and biochemical traits

The heterosis estimates over better parent (heterobeltiosis) for the studied traits are furnished in **Table 6**. The hybrid combinations Giza177 × Sakha108 and Giza177 × Sakha106 were the best combinations for the RWC trait under both conditions as they recorded the highest significant and positive (desirable) heterosis estimates over better parents for this trait. In comparison, the hybrids recorded the highest significant positive heterotic effects relative to the better parent for the trait of proline content (Puebla × Sakha106 and Hispagran × Sakha108 under normal conditions and Hispagran × Sakha106 under water deficit conditions). Concerning the activities of antioxidant enzymes, the highest significant and desirable values of heterobeltiosis were obtained in the crosses Puebla × Sakha106 and Hispagran × Sakha106 for CAT enzyme activity and in the crosses Hispagran × Sakha106 and Hispagran × Sakha108 for APX enzyme activity, under both conditions. Regarding total chlorophyll content, the hybrid combination Giza177 × Sakha107 under both irrigation treatments, followed by Puebla × Sakha106 under normal irrigation and Giza177 × Sakha106 under water shortage conditions, was the best hybrid which showed the highest heterosis estimates over better parent for total chlorophyll content.

#### 3.5.2 Yield traits

The data presented in **Table 6** confirmed that the crosses Puebla × Sakha106, Puebla × Sakha107, Hispagran × Sakha106, and Hispagran × Sakha108 under both conditions were the best combinations for the most studied yield traits as they showed the highest significant and negative (desirable) heterosis relative to the better parent for earliness and plant height; this suggested the possibility of developing early maturity and short plant stature hybrids from these cross combinations. For grain yield/plant, there were nine crosses under normal conditions. Eight crosses under water deficit conditions recorded significant and positive (desirable) values of heterosis, but Puebla × Sakha107 was the best hybrid under both irrigation conditions, followed by Giza177 × Sakha107 under normal conditions. However, Puebla × Sakha106 under water deficit conditions gave the highest significant positive (desirable) values of heterosis for single plant grain yield. Only one hybrid (Giza177 × Sakha107) showed significant and positive heterosis relative to the better parent for spikelet fertility under normal irrigation. On the other hand, the hybrids Hispagran × Sakha108 and Hispagran × Sakha106, followed by Giza177 × Sakha107 and Giza177 × Sakha106, had highly significant positive heterotic effects relative to the better parent for this trait under water deficit conditions. Regarding the 1,000-grain weight, the highest positive significant better parent based on heterosis was estimated for Hispagran × Sakha106 under normal and water deficit conditions, followed by Giza177 × Sakha107, Puebla × Sakha106, and Hispagran × Sakha108 under normal conditions and Sakha105 × Sakha107 and Hispagran × Sakha108 under water deficit conditions.

### 3.6 Estimates of genetic parameters

Estimations of genetic variance components, heritability, and contribution of the lines, testers, and line × tester for all the studied traits under normal and water deficit conditions were recorded (**Table 7**). The findings demonstrated that, under both irrigation conditions, the dominance genetic variance (σ^2^D) was greater than the additive genetic variance (σ^2^A) in controlling the inheritance of all traits under study. The importance of additive and non-additive gene effects in these traits was confirmed by the high estimates of heritability in a broad sense (h^2^_b_) found for all of the studied traits. These estimates ranged from 77.13% to 99.01% for the traits of proline content and CAT enzyme activity, respectively, under normal conditions. However, heritability estimates in the narrow sense (h^2^_n_ %) were relatively moderate for the traits of CAT activity under normal conditions (24.32%), RWC and total chlorophyll content under water deficit conditions (24.00% and 22.81%, respectively), and the 1,000-grain weight under both conditions (25.30% and 26.93%, respectively), while the estimates were low for the other traits under both conditions. It is evident that the lines played an important role in all studied traits under both irrigation conditions, ranging from 43.61% to 78.24% for CAT and RWC, respectively, under water deficit conditions; this indicates the predominance of paternal effects as demonstrated by the lines for these traits.

**Table 7:** Estimates of genetic parameters for physiological, biochemical, and yield-related (and its components) traits under normal watering and water deficit conditions.

Genotypes	RWC (%)		Proline (mg g^−1^ FW)		CAT (unit mg^−1^ protein)		APX (unit mg^−1^ protein)		Total chlorophyll (mg g^−1^ FW)	
	N	W. D	N	W. D	N	W. D	N	W. D	N	W. D
**Additive variance (σ^2^A)**	7.113	14.797	0.003	0.004	0.90	0.61	0.74	1.09	0.06	0.11
**Dominant variance (σ^2^D)**	38.497	45.153	0.009	0.014	2.78	2.25	3.55	4.63	0.27	0.37
**Environmental variance (σ^2^E)**	2.611	1.707	0.003	0.002	0.04	0.03	0.09	0.07	0.01	0.01
**Broad sense heritability h^2^_b_, %**	94.58	97.23	77.13	91.18	99.01	98.85	98.02	98.82	98.42	98.93
**Narrow sense heritability h^2^_n_, %**	14.75	24.00	20.82	18.45	24.32	20.93	16.99	18.85	18.73	22.81
**Contribution of lines, %**	63.67	78.24	70.36	64.36	49.07	43.61	44.96	57.48	44.90	59.97
**Contribution of testers, %**	8.51	2.22	10.19	12.20	31.42	34.25	29.29	18.53	31.05	19.40
**Contribution of line × tester, %**	27.83	19.54	19.45	23.44	19.51	22.14	25.74	23.98	24.06	20.63
Genotypes	Days to heading (days)		Plant height (cm)		Grain yield/plant (g)		Spikelet fertility (%)		1,000-grain weight (g)	
	N	W. D	N	W. D	N	W. D	N	W. D	N	W. D
**Additive variance (σ^2^A)**	5.51	4.97	3.67	2.00	3.45	1.89	0.40	0.47	0.59	0.79
**Dominant variance (σ^2^D)**	26.11	26.30	24.17	13.56	17.93	7.99	2.27	2.45	1.21	1.69
**Environmental variance (σ^2^E)**	2.27	1.49	2.36	1.64	2.34	1.72	0.35	0.35	0.53	0.45
**Broad sense heritability h^2^_b_, %**	93.31	95.45	92.18	90.47	90.12	85.20	88.37	89.29	77.42	84.64
**Narrow sense heritability h^2^_n_, %**	16.27	15.16	12.15	11.65	14.55	16.29	13.30	14.48	25.30	26.93
**Contribution of lines, %**	61.83	64.77	67.67	58.03	69.06	70.85	67.24	62.08	70.62	77.11
**Contribution of testers, %**	12.23	7.79	1.42	10.55	3.42	4.28	3.89	10.42	13.23	6.86
**Contribution of line × tester, %**	25.94	27.45	30.92	31.42	27.51	24.87	28.87	27.50	16.15	16.03

N, normal condition; W. D, water deficit condition.

On the contrary, the contributions of the tester were lower than the contributions of the lines for all traits under study under normal and water deficit conditions, as the maximum value of the testers’ contribution was 34.25% for CAT enzyme activity under water deficit conditions. In contrast, the minimum value was 1.42% and recorded by plant height under normal conditions. For proline content and CAT activity under normal conditions, RWC under water deficit conditions, and 1,000-grain weight under both conditions, the contribution of the line × tester interaction was relatively moderate for all physiological and yield traits under both conditions.

### 3.7 Correlation analysis

**Figure 2:**
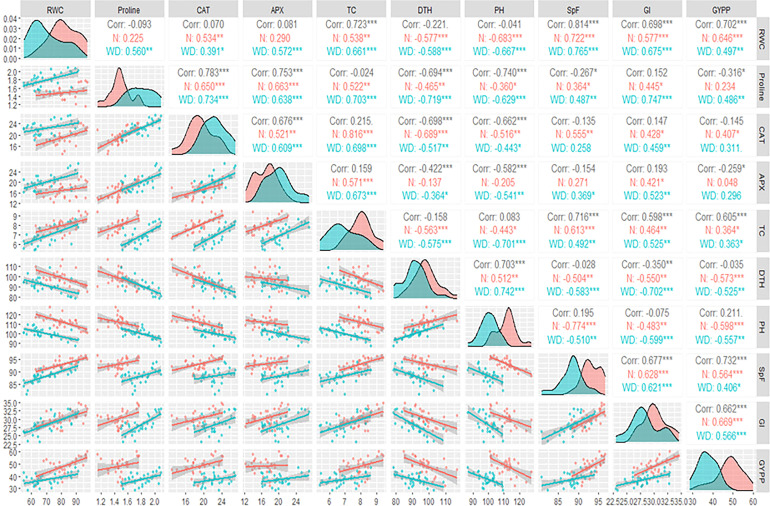
Pearson correlation matrix among the studied traits under normal and water deficit conditions.*, ** and *** indicate p< 0.05, p<0.01 and p<0.001.

The upper triangle showed the Pearson coefficient correlation matrix among the studied traits under normal, water deficit, and both conditions (**Figure 2**). Under both conditions, PH and the number of days to 50% heading (DTH) were negatively and moderately significantly correlated to grain yield per plant (GYPP) under normal and water deficit conditions. On the other hand, the weight of 1,000 grains (GI), spike fertility (SpF), and RWC were positively and moderately significantly correlated to GYPP. In the same context, total chlorophyll (TC) was positively but weakly significantly correlated to GYPP. Proline was positively and significantly correlated to GYPP under water deficit conditions.

On the one hand, APX activity was not correlated to GYPP under either normal or water deficit conditions. On the other hand, CAT activity was positively and moderately significantly correlated to GYPP only under normal conditions. Most of the other correlations among the studied traits were moderate in strength and significant. The diagonal illustrated the density plots of the studied traits (**Figure 2**). The area refers to the highest density of the values under the curve. From the density plots, it was observed that all of the studied traits had different peaks under normal and water deficit conditions; this means that the values of those traits under normal conditions were concentrated at higher values than water deficit conditions except for proline, CAT, and APX enzymes. It can be concluded from the density plots that the impact of water deficit on the studied traits was mainly on the magnitude of that trait and its density.

### 3.8 Cluster analysis

**Figure 3:**
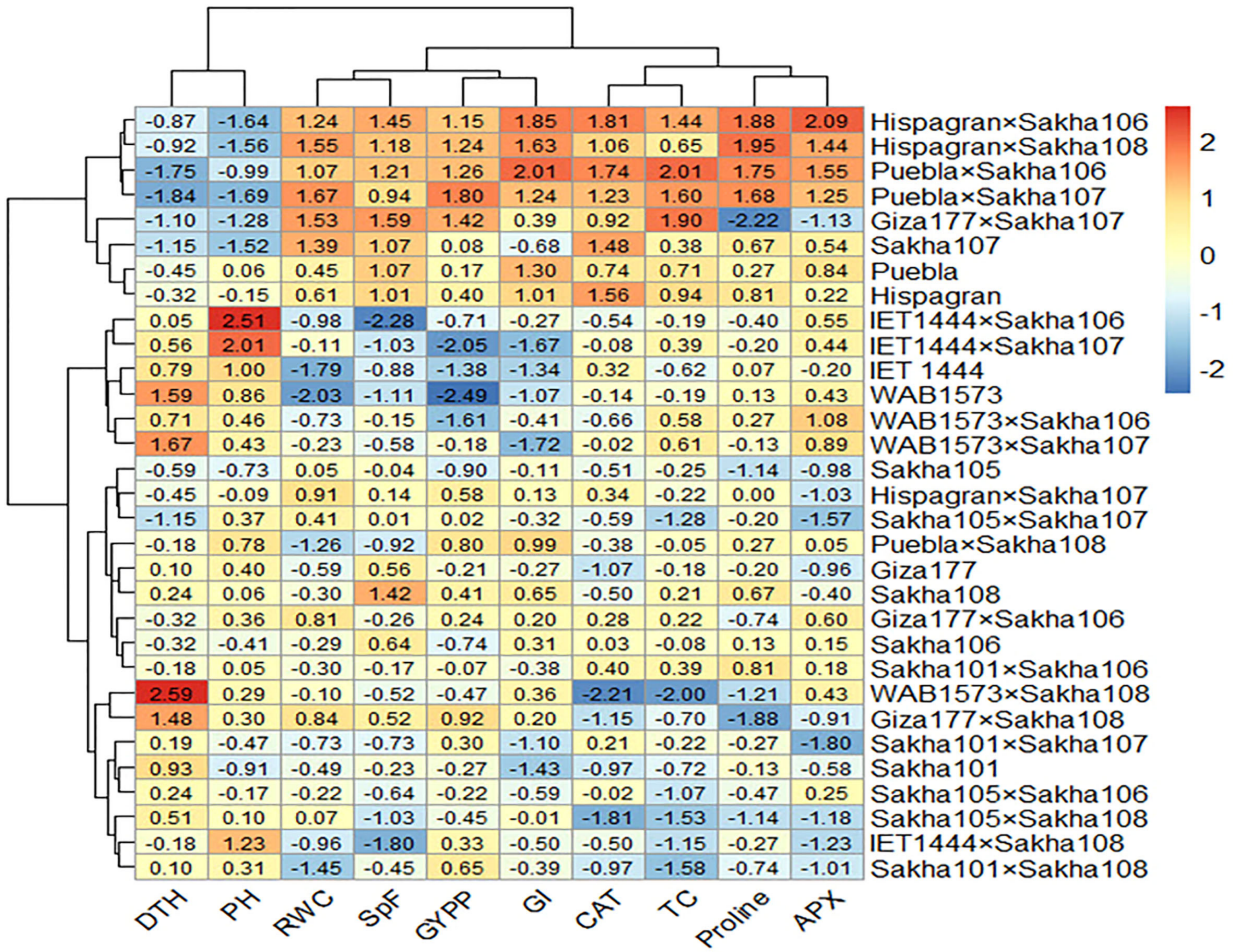
Heatmap showing the relationship between genotypes and the studied traits under normal conditions based on standardized data.

**Figure 4:**
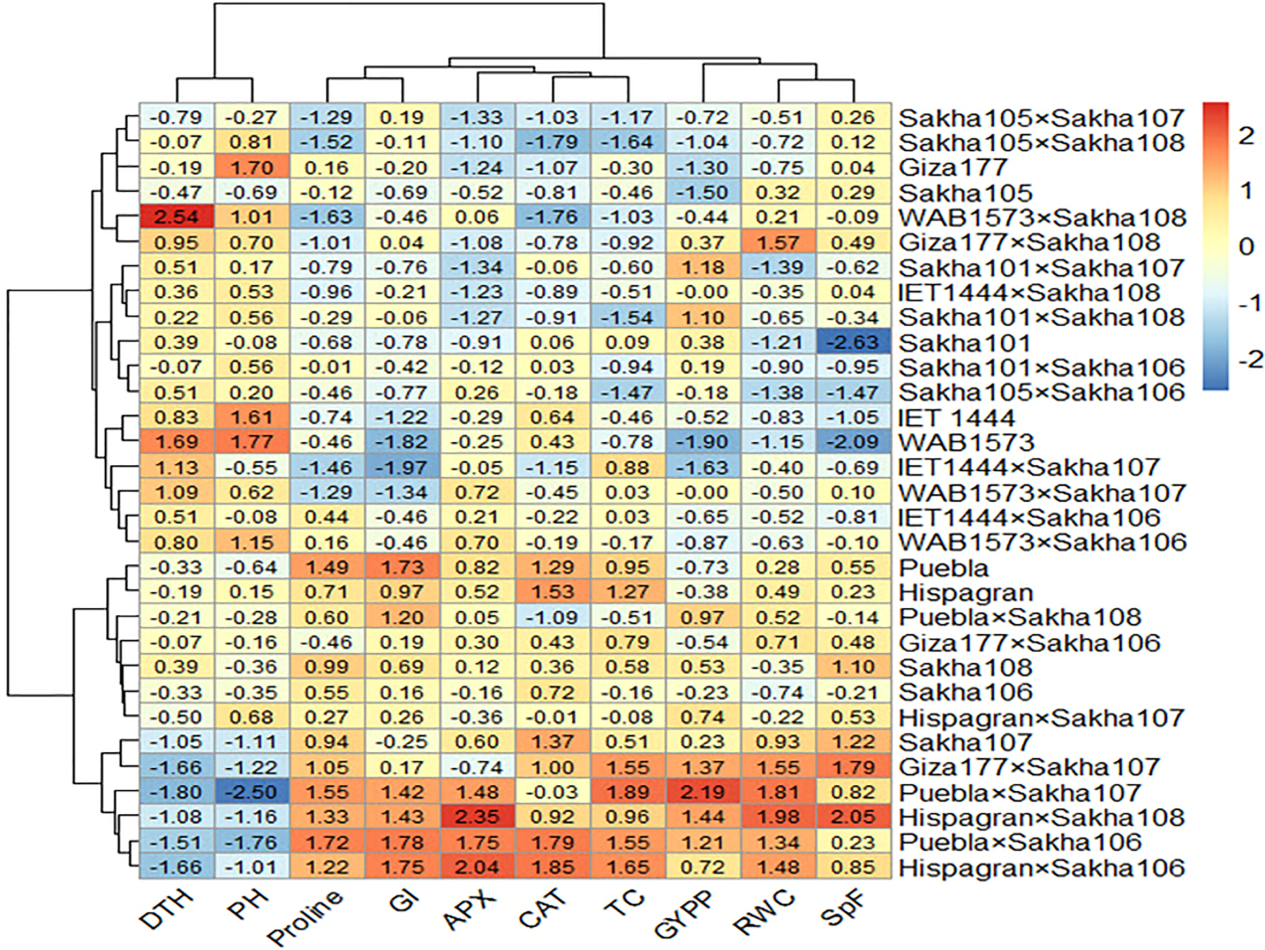
Heatmap showing the relationship between genotypes and the studied traits under water deficit conditions based on standardized data.

All the genotypes were grouped into two clusters under normal and water deficit conditions (**Figure 1**), where the averages of the studied traits were shown (**Table 8**). The structure of the clusters was changed when the genotypes were subjected to water deficit conditions except for the genotypes Sakha106, Sakha108, Giza177 × Sakha106, Hispagran × Sakha107, and Puebla × Sakha108, which moved from cluster 1 under normal conditions to cluster 2 under water deficit conditions because they were more tolerant to water deficit expressed by all the studied traits than the other genotypes of their cluster. Heatmap cluster analysis showed the relationship between the genotypes and the studied traits depending on standardized data using a color scale under normal and water deficit conditions (**Figures 3**, **4**). The red color represents high values of the studied traits, while the blue color represents low values. Genotypes Hispagran × Sakha106, Hispagran × Sakha108, Puebla × Sakha106, Puebla × Sakha107, and Giza177 × Sakha107 showed the highest GYPP under normal and water deficit conditions. On the other hand, genotypes IET1444, WAB1573, IET1444 × Sakha107, and WAB1573 × Sakha106 were the lowest in GYPP under normal conditions, while WAB1573, IET1444 × Sakha107, Sakha 105, Giza177, and Sakha105 × Sakha108 were the lowest in GYPP under water deficit conditions.

**Table 8:** Averages of the studied traits for the two clusters under normal and water deficit conditions.

Clusters	RWC	Proline	CAT	APX	TC	DTH	PH	GYPP	SpF	GI
Normal conditions										
Cluster1	93.20	1.61	22.80	19.00	8.87	90.60	105.00	54.40	95.80	32.60
Cluster2	78.00	1.44	17.90	16.10	7.73	100.80	114.00	46.10	92.30	29.40
Water deficit conditions										
Cluster1	78.30	1.96	24.30	22.10	7.68	86.00	96.10	39.80	90.30	30.60
Cluster2	64.10	1.67	20.90	18.60	6.34	95.10	103.00	35.30	86.80	26.50

From the heatmap cluster analysis, it is clear that the highest values of GYPP were mainly explained by the highest values of all the studied traits except for PH and DTH under normal and water deficit conditions. Also, lower GYPP was mainly due to lower values of GI, SpF, and RWC, where the blue color in GYPP was associated with blue in SpF under normal conditions. In contrast, under water deficit conditions, lower GYPP was mainly due to lower values of GI in WAB1573 and IET1444 × Sakha107, lower values of APX and CAT in Giza177 and Sakha105 × Sakha108, and lower values of GI, APX, and CAT in Sakha105.

## 4 Discussion

Rice is considered one of the major stable foods for more than a third of humans worldwide (Dianga et al., 2020). In Egypt, rice is a strategic and essential crop regarding food security. Population growth was the most influential driver for the development of rice productivity to satisfy the rising demand for rice. However, environmental stress factors may exacerbate the problem by increasing the frequency and intensity of such abiotic stresses (Awad-Allah, 2020; El-Hendawy et al., 2020; Desoky et al., 2021). Water deficiency has been described as the most acute and major limiting factor for rice production in rainfed settings. Plants have developed different adaptive mechanisms through evolution to survive and cope with adverse environments (Gaballah et al., 2022). To mitigate the reduction in rice yield, there is a continual need for the enhanced creation of climate-resilient varieties (Kumari et al., 2022). To this end, breeders should apply both old and developing strategies to obtain rice genotypes with tolerance to water scarcity stress; this is an economically viable and sustainable strategy for enhancing rice production to meet the rising demand (Premkumar et al., 2017; Peterson et al., 2018; Van Tran et al., 2021). We examined the mode of gene action, the magnitude of heterosis, and the general and specific combining ability for yield, contributing traits, and some physiological and biochemical traits in some rice lines, testers, and their F_1_ crosses under water deficit stress in order to identify the most desirable genotypes for rice breeding programs under water shortage conditions.

According to the analysis of variance, the highly significant mean squares due to the genotypes and their partitions indicate a high degree of genetic diversity among the used genotypes, indicating that the germplasm used in the study possessed a high degree of genetic diversity. The importance of parents identifying the most desirable genotypes was prevalent for all attributes. Therefore, the genotypes evaluated can enhance grain yield and other studied attributes in crops susceptible to water scarcity. Similar results were reported (Ganapati et al., 2020; Gaballah et al., 2022).

The average performance of the tested traits varied according to genotype and irrigation circumstances. Water deficiency inhibits water uptake by the leaves from the root system (Arjenaki et al., 2011). As a result, it reduced water-holding capacity and stomatal movement, limiting chlorophyll synthesis, CO_2_ influx to the leaves, and photosynthesis. In addition, ROS accumulation promotes chlorophyll breakdown, chloroplast destruction, and a decrease in photosystem II activity (Osakabe et al., 2014). Breeders prefer the highest mean values for all physiological and yield parameters, with the exception of days to heading and plant height, for which the lowest mean values were preferred. Except for proline content and antioxidant enzyme activity (CAT and APX), which rose dramatically, water scarcity caused considerable reductions in all examined characteristics relative to normal irrigation. Tolerance to drought stress in higher plants is associated with strong antioxidant systems and, thus, a relatively higher ability to scavenge the ROS from drought-induced oxidative stress. Plant response to drought is complex, altering many physiological processes in response to these adverse conditions (Kumar et al., 2020). Water scarcity caused considerable reductions in all examined characteristics relative to normal irrigation, except for proline content and antioxidant enzyme activity, which rose dramatically to reduce ROS-induced oxidative damage.

In contrast, proline synthesis and the activation of CAT and APX enzyme activities were considerably higher in stressed rice plants than in non-stressed plants. Yield-contributing characteristics are the ultimate consequence of physiological processes occurring at various stages of development; consequently, these traits increase dramatically to reduce ROS-induced oxidative damage. Based on the per se performance, the genotypes with the lowest mean values for earliness and short plant stature and the highest mean performance for all remaining attributes have been considered superior parents (Herwibawa et al., 2019; Al Azzawi et al., 2020).

The combining capacity was investigated to discover genotypes with high genetic potential for creating cross combinations with desired traits and to examine the action of genes involved in trait expression. From a genetic standpoint, the GCA assesses additive and additive × additive gene activity. Hence, selecting parents with favorable GCA effects was essential for a successful breeding program, particularly hybrid breeding. Simultaneously, the SCA of the crosses was the evaluation and comprehension of the effect of non-additive gene action on a trait. A trait’s non-additive gene action denotes the selection of a hybrid combination. Large positive values of GCA and SCA impacts would be of interest to breeders for all tested attributes, with the exception of days to heading and plant height, where high negative estimations would be advantageous for enhancing these traits in breeding strategies. The values of σ²GCA variance were less than the value of σ²SCA variance, and the ratio of σ²GCA/σ²SCA was less than unity for all the studied yield and physiological traits under both treatments. In addition, the dominance genetic variance (σ^2^D) was greater than σ^2^A in controlling the inheritance of all studied traits under both irrigation conditions, revealing that the non-additive gene effects played a significant role in the genetic expression of these traits.

Consequently, hybridization is possible for features governed by non-additive gene activity, followed by selection in later generations. However, the lines Puebla and Hispagran had the highest significant positive values for the majority of physiological and yield traits, as well as the highest significant negative and desirable GCA effects for earliness and short stature under both irrigation conditions, indicating that Puebla and Hispagran were the best general combiners compared with other parents for the majority of physiological and yield traits under normal and water deficit conditions. The crosses Puebla × Sakha107 and Hispagran × Sakha108 recorded the highest significant and desirable SCA effects for the studied physiological traits under both irrigation conditions, whereas the crosses Giza177 × Sakha107, Hispagran × Sakha108, and IET1444 × Sakha108 also showed the highest significant and desirable SCA estimates for the studied yield traits under both normal and water deficit conditions. In most cases, parents with a high or low GCA value were involved in Hispagran and had the highest significant positive values for most physiological and yield traits. Notable is the fact that crosses with strong SCA effects for grain yield also exhibited high SCA effects for one or more yield component characteristics. Therefore, these crossings could be proposed to improve the respective qualities obtained by including water deficit tolerance in a rice hybrid breeding program. Earlier researchers have also confirmed the effects of additive and non-additive genes and their benefits on generating hybrid rice types (Singh and Kumar, 2019; El-Mowafi et al., 2021; Abd El-Aty et al., 2022a).

Heterosis is particularly essential because it is anticipated that the hybrid to be introduced would surpass the existing superior local hybrid variety. High positive heterosis values would interest breeders for most examined traits; however, high negative heterosis values would be advantageous for days to heading and plant height. The results illustrated that the hybrid combinations Puebla × Sakha106, Puebla × Sakha107, Hispagran × Sakha106, and Hispagran × Sakha108 recorded the highest significant values for the studied physiological and yield traits, in addition to the highest significant and negative (desirable) estimates of heterobeltiosis for the traits of days to 50% flowering and plant height; this means that these crosses, with highly significant heterosis estimates, might have the desirable genes for earliness and short plant stature besides high yielding under both irrigation conditions. These cross combinations could develop early maturity genotypes because early maturing hybrids show lodging resistance; this suggested that these crosses could be utilized as good cross combinations for improving such characteristic in the hybrid rice breeding program. These results are in close agreement with the findings of Gaballah et al. (2022) and Premkumar et al. (2017).

The results of genetic parameter analysis revealed that heritability h^2^_b_ values were higher than the h^2^_n_ values for all the studied characteristics under normal and water deficit conditions. Selection for the desired genotypes based on phenotype performance may be effective for yield component traits. The lines contributed significantly more than the testers and their interactions for all yield traits under normal and water deficit conditions. These results are in agreement with those obtained by Abd El-Hadi et al. (2013) and Hasan et al. (2015).

## 5 Conclusion

Water deficit stress significantly decreased relative water content, total chlorophyll content, grain yield, and yield attributes. In contrast, it significantly increased proline content and antioxidant enzyme activities (CAT and APX) compared with normal irrigation conditions. The parental genotypes Puebla and Hispagran were identified as good combiners for most physiological and biochemical traits under study in addition to earliness, shortness, grain yield, and 1,000-grains weight traits under both irrigation conditions. Additionally, the cross combinations Puebla × Sakha107, Hispagran × Sakha108, and Giza177 × Sakha107 were the most promising combinations demonstrating substantial and desirable specific combining ability effects on all the tested traits, which suggested that they could be considered for use in rice hybrid breeding programs.

## Data availability statement

The original contributions presented in the study are included in the article/**Supplementary Material**. Further inquiries can be directed to the corresponding author.

## Author contributions

All authors have contributed equally to the research and analysis of the various results and sections within the review. All have corrected and modified the different versions of the manuscript as prepared by the corresponding and senior authors. All authors read and approved the final manuscript.

## Supplementary material

Supplementary Figure 1
**(A, B)** the weather data (rain in mm, the average temperature in °C)- the weather data (radiation in MJ/m2).
