# Supplementary figures and images for: Mode of gene action and heterosis for physiological, biochemical, and agronomic traits in some diverse rice genotypes under normal and drought conditions
Source: Front Plant Sci. 2023 Mar 31;14:1108977. doi: 10.3389/fpls.2023.1108977 (PMC10103692; doi:10.3389/fpls.2023.1108977)

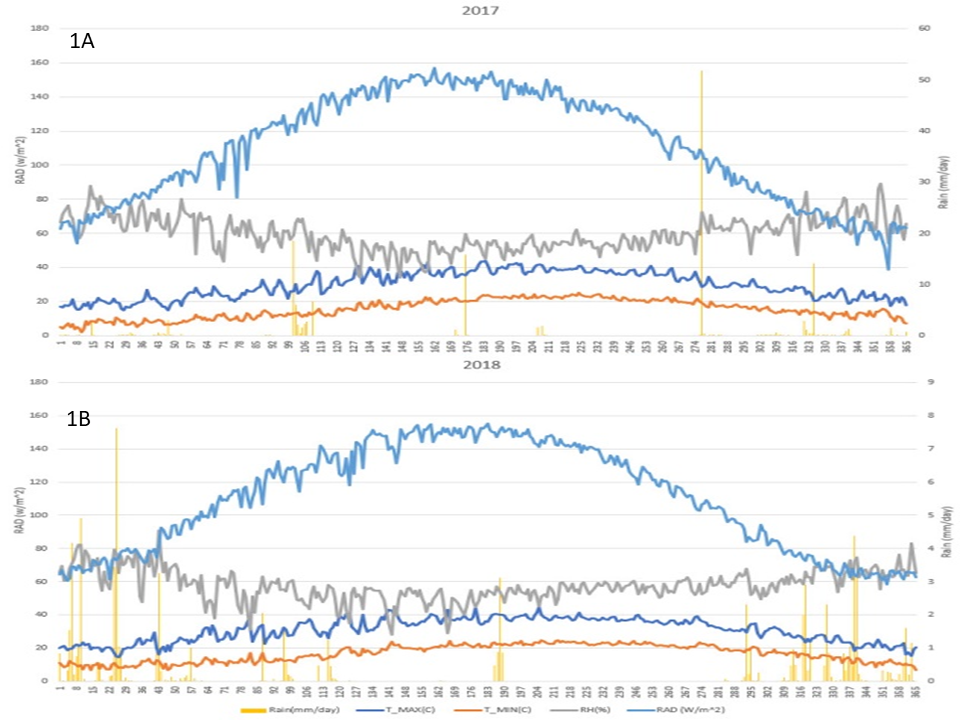

Supplement: Supplementary Figure 1 — (A, B) the weather data (rain in mm, the average temperature in °C)- the weather data (radiation in MJ/m2). [file Image_1.tif]
