# Supplementary material for: Mode of gene action and heterosis for physiological, biochemical, and agronomic traits in some diverse rice genotypes under normal and drought conditions
Source: Front Plant Sci. 2023 Mar 31;14:1108977. doi: 10.3389/fpls.2023.1108977 (PMC10103692; doi:10.3389/fpls.2023.1108977)
Supplement: Supplementary file 2 [file Table_1.docx]

**Table S1:** Some physical and chemical properties of the experimental soil before sowing in the first season.

| **Soil properties** | Kafr EL-Shiekh |
| --- | --- |
| Mechanical:  Clay % | 56.00 |
| Silt % | 32.00 |
| Sand % | 12.00 |
| Texture | Clayey |
| Chemical: |  |
| Organic Matter (O.M)% | 1.50 |
| pH(1:2.5 soil suspension) | 8.44 |
| Ec (ds.m^-1^) | 3.34 |
| Total N (ppm) | 430.50 |
| Available P (ppm) | 12.00 |
| Available K (ppm) | 432 |
